# Efficacy of Indian polyvalent snake antivenoms against Sri Lankan snake venoms: lethality studies or clinically focussed *in vitro* studies

**DOI:** 10.1038/srep26778

**Published:** 2016-05-27

**Authors:** Kalana Maduwage, Anjana Silva, Margaret A. O’Leary, Wayne C. Hodgson, Geoffrey K. Isbister

**Affiliations:** 1 Clinical Toxicology Research Group, University of Newcastle, NSW, Australia; 2 South Asian Clinical Toxicology Research Collaboration (SACTRC), Faculty of Medicine, University of Peradeniya, Peradeniya, Sri Lanka; 3 Department of Pharmacology, Monash Venom Group, Monash University, Clayton, Victoria, Australia; 4 Department of Parasitology, Faculty of Medicine and Allied Sciences, Rajarata University of Sri Lanka, Anuradhapura, Sri Lanka

## Abstract

*In vitro* antivenom efficacy studies were compared to rodent lethality studies to test two Indian snake antivenoms (VINS and BHARAT) against four Sri Lankan snakes. *In vitro* efficacy was tested at venom concentrations consistent with human envenoming. Efficacy was compared statistically for one batch from each manufacturer where multiple vials were available. In binding studies EC_50_ for all VINS antivenoms were less than BHARAT for *D. russelii* [553 μg/mL vs. 1371 μg/mL;p = 0.016), but were greater for VINS antivenoms compared to BHARAT for *N. naja* [336 μg/mL vs. 70 μg/mL;p < 0.0001]. EC_50_ of both antivenoms was only slighty different for *E. carinatus* and *B. caeruleus*. For procoagulant activity neutralisation, the EC_50_ was lower for VINS compared to BHARAT - 60 μg/mL vs. 176 μg/mL (p < 0.0001) for Russell’s viper and 357 μg/mL vs. 6906*μ*g/mL (p < 0.0001) for Saw-scaled viper. Only VINS antivenom neutralized *in vitro* neurotoxicity of krait venom. Both antivenoms partially neutralized cobra and didn’t neutralize Russell’s viper neurotoxicity. Lethality studies found no statistically significant difference in ED_50_ values between VINS and BHARAT antivenoms. VINS antivenoms appeared superior to BHARAT at concentrations equivalent to administering 10 vials antivenom, based on binding and neutralisation studies. Lethality studies were inconsistent suggesting rodent death may not measure relevant efficacy outcomes in humans.

Snake envenoming is recognised as a major global health issue with large numbers of deaths and cases of envenoming occurring each year in tropical and sub-tropical regions of the world^1^. Although antivenom is the accepted treatment for snake envenoming, there is a shortage of antivenoms in Africa and Asia and in some cases this has led to the use of antivenom made in one country being used in a different country, that may not be effective against snake venoms in that geographical region^2^,^3^,^4^,^5^. Over the last two decades there have been concerns about the efficacy of some antivenoms and whether sufficient doses are being used^6^,^7^. In some cases this has led to changes in dosing based on such concerns and in others the development of new antivenoms^8^.

A particular problem with developing antivenoms is testing their efficacy and more importantly the potential efficacy in humans, including appropriate dosing. The World Health Organisation (WHO) recommends the use of median lethal dose (LD_50_) and median effective dose (ED_50_) for assessing the efficacy of antivenoms^9^. However, there are significant animal ethical considerations because of the numbers of animals that are required for these tests, and there are concerns about extrapolating the death of a rodent to clinical effects in humans.

Although the exact mechanism of venom-induced death is unclear in rodents, death may be due to the effects of post-synaptic neurotoxins or early cardiovascular collapse^10^. In contrast, pre-synaptic neurotoxins are important in human envenoming^11^, as well as toxins that cause coagulopathy, myotoxicity and nephrotoxicity. For example, a recent study has demonstrated that rodent plasma is highly resistant to procoagulant toxins that are highly relevant for human envenoming^12^. This means that the use of death of a rodent as a test of efficacy for snake venoms that cause coagulopathy is problematic.

Other *in vitro* and *in vivo* investigations are available to test the efficacy of antivenom to neutralise relevant pathophysiological effects induced by snake venoms and may provide more clinically useful information^13^,^14^,^15^,^16^. For snakes that cause coagulopathy in humans it would seem more appropriate to test the efficacy of antivenom to neutralise procoagulant venom activity in human plasma, rather than the efficacy of antivenom in preventing death in a rodent. The fields of venomics and antivenomics are also beginning to improve our understanding of the different composition of venoms and their interaction with antivenoms^17^,^18^. However, it will be important to ensure that antivenoms are tested in functional studies of clinically relevant venom effects.

Currently all antivenom used in Sri Lanka is supplied from India and there are limited data regarding the efficacy of Indian antivenoms against Sri Lankan snakes^19^. Concerns were raised about the effectiveness of Indian polyvalent antivenoms in Sri Lanka in 2012 and there were sufficient concerns about the efficacy of antivenom for the treatment of Russell’s viper (*Daboia russelii*) envenoming that the Government requested independent testing of the antivenoms. Treating doctors had observed an increased number of hospital deaths and reactions to the antivenom.

The aim of this study was to investigate the efficacy of two different Indian polyvalent antivenoms comparing a range of *in vitro* studies to classical lethality studies. In doing so we aimed to test the ability of the antivenoms to neutralise 1) the procoagulant effect of two viper venoms–Russell’s viper (*D. russelii*) and the saw-scaled viper (*Echis carinatus*); 2) neurotoxic effect of three snake venoms–common krait (*Bungarus caeruleus*), common cobra (*Naja naja*) and Russell’s viper (*D. russelii*); comparing these to LD_50_/ED_50_ studies.

## Results

All *in vitro* experiments were done at venom concentrations measured in cases of human envenoming which was 1.7 μg/ml for *D. russelii*, 0.5 μg/ml for *E. carinatus*, 1.4 μg/ml for *N. naja* and 0.15 μg/ml for *B. caeruleus*. Multiple batches of antivenoms from both VINS Bioproducts Limited and BHARAT Serum and Vaccines Limited were tested and compared. To statistically compare the efficacy of the two antivenoms one batch from each manufacturer was compared where multiple vials were available–VINS (01011/10/11; 2010) and BHARAT (A5311006; 2011Jan).

### Protein quantification

The median dry powder weight (mg) of antivenom, the median percentage of protein per mg of antivenom and the median dry powder weight (mg) of protein per antivenom vial were measured (Table 1). All batches of VINS antivenom had a higher protein concentration than the batch of BHARAT antivenom tested (Table 1). The mean protein content of 10 vials of VINS antivenom from the same batch (01011/10–11) was 198 mg (Standard Deviation [SD]: 28 mg), which was significantly higher than the mean protein content of 10 vials of BHARAT antivenom from the same batch (A5311006), 98 mg SD: 29 mg; p < 0.0001; unpaired t-test).

### Venom-antivenom binding studies

**Figure 1:**
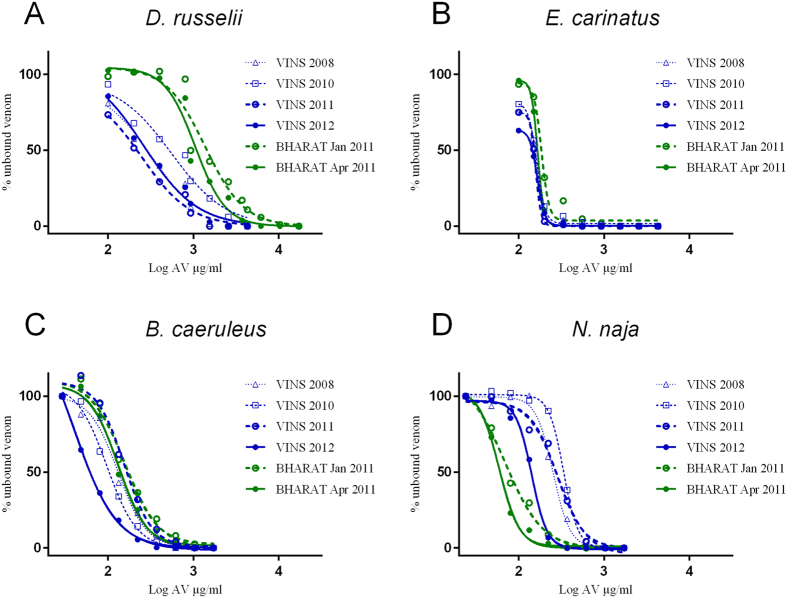
Plots of percent free (unbound) venom versus the logarithm of the antivenom concentration for six batches of antivenom, four VINS and two BHARAT antivenoms showing the binding capacity for (**A**) *D. russelii*, (**B**) *E. carinatus*, (**C**) *N. naja* and (**D**) *B. caeruleus* venoms.

The median effective concentration (EC_50_) for antivenom binding was the concentration of antivenom that bound 50% of free venom antigens at clinically relevant venom concentrations. The EC_50_ for both antivenoms are given in Table 2 and the venom-antivenom binding curves are shown graphically in Fig. 1 and Supp Figure 1. The EC_50_ values of *D. russelii* venom for all VINS antivenoms were less than for BHARAT antivenoms, and was 553 μg/mL (95% confidence intervals [CI]: 237–1289 μg/mL) for VINS 2010 compared to 1371 μg/mL (95% CI: 956–1965 μg/mL) for BHARAT 2011, which was statistically significantly different (p = 0.016). The EC_50_ values for *N. naja* venom of all VINS antivenoms were greater than all BHARAT antivenoms, 336 μg/mL (95% CI: 325–347 μg/mL) for VINS 2010 compared to 70 μg/mL (95% CI: 53–91 μg/mL) for BHARAT 2011 (p < 0.0001). The EC_50_ values for *E. carinatus* and *B. caeruleus* venoms of VINS and BHARAT antivenoms did not appear to differ markedly (Table 2, Fig. 1), but was statistically significantly different when comparing the two batches for *B. caeruleus* [97 μg/mL (95% CI: 85–110 μg/mL) for VINS versus 157 μg/mL (95% CI: 121–205 μg/mL) for BHARAT; p = 0.002], but not for *E. carinatus* [165 μg/mL (95% CI: 156–172 μg/mL) for VINS versus 187 μg/mL (95% CI: 168–208 μg/mL) for BHARAT; p = 0.053] (Supp Figure 1).

### Neutralization of procoagulant activity of D. russelii and E. carinatus venoms

**Figure 2:**
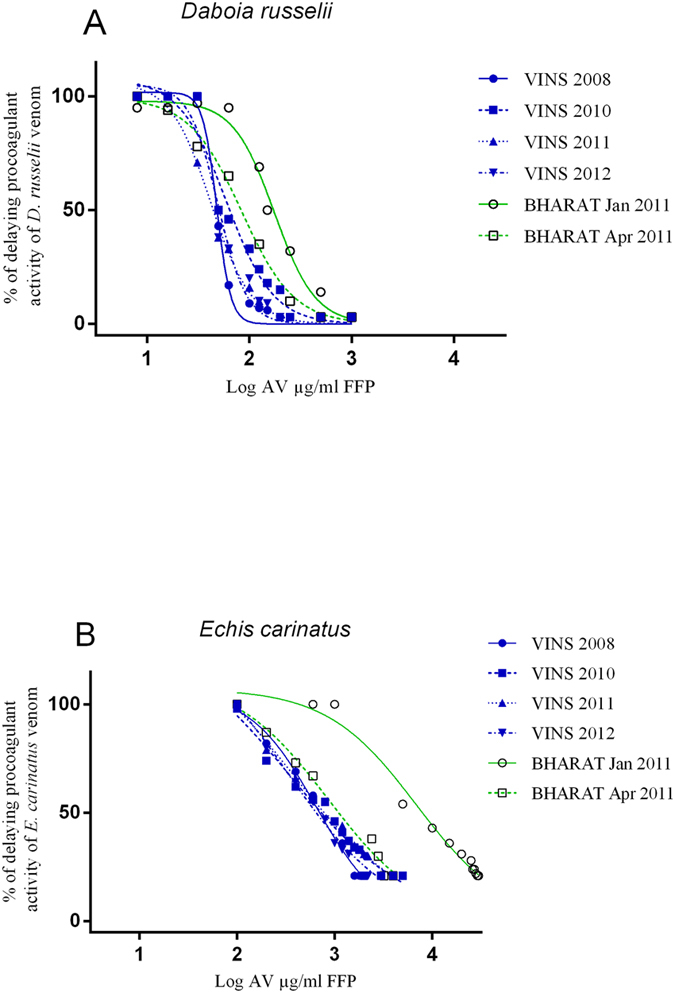
Effect of VINS and BHARAT antivenoms on delaying the procoagulant activities of 1.7 μg/mL of *D. russelii* (**A**), 0.5 μg/mL of *E. carinatus* (**B**) venom on human plasma.

VINS antivenoms were able to neutralise the procoagulant activity of both venoms at much lower antivenom concentrations than BHARAT antivenom, based on lower median effective concentrations against procoagulant activity (EC_50_; Table 3, Fig. 2, Supp Figure 2). The EC_50_ against procoagulant activity of *D. russelii* for VINS was 60 μg/mL (95% CI: 44–81 μg/mL) compared to 176 μg/mL (95% CI: 149–207 μg/mL) for BHARAT, which was significantly different (p < 0.0001). Much higher concentrations were required by both antivenoms to neutralise the procoagulant effect of *E. carinatus* despite there being lower concentrations of venom present. (Table 3, Fig. 2). The EC_50_ against procoagulant activity of *E. carinatus* for VINS was 357 μg/mL (95% CI: 87–1458 μg/mL) compared to 6906 μg/mL (95% CI: 4859–9817 μg/mL) for BHARAT, which was significantly different (p < 0.0001).

### Neurotoxicity of B. caeruleus, N. naja and D. russelii venoms

**Figure 3:**
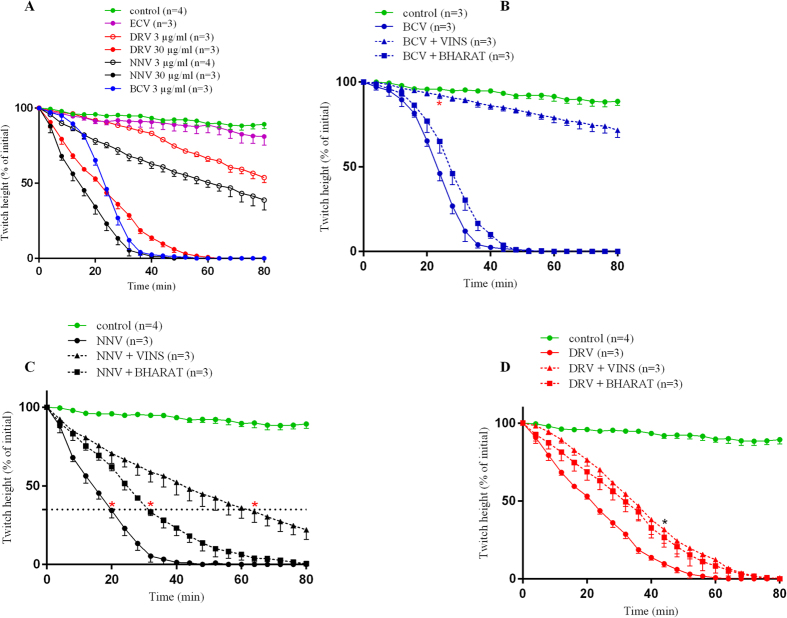
Plots of twitch height (mean ± standard error of the mean [SEM]) versus time - (**A**) The neurotoxic effect of *B. caeruleus, N. naja, D. russellii* and *E. carinatus* venoms alone on indirect twitches of the chick biventer nerve–muscle preparation; and the effect of prior administration (10 min) of VINS and BHARAT antivenoms (five times the recommended dose) to (**B**) *B. caeruleus* (3 μg/ml) venom (*Twitch height of BCV + VINS after 24 min is significantly different from the BCV and BCV + BHARAT and not different from the controls: p < 0.05, one way ANOVA followed by Bonferroni’s post-hoc test), (**C**) *N. naja* (30 μg/ml) venom (*Time taken for the maximum drop of twitch height in all tissues in NNV + VINS, i.e. by 65% is significantly prolonged compared to similar twitch inhibition in NNV and NNV + BHARAT: p < 0.05, Kruskal-Wallis test followed by Dunn’s multiple comparison test), and (**D**) *D. russelii* (30 μg/ml) venom on indirect twitches of the chick biventer nerve–muscle preparation (*Twitch height of DRV + VINS and DRV + BHARAT after 44 min is not different from DRV while all above are different from the controls: p < 0.05, one way ANOVA followed by Bonferroni’s post-hoc test). BCV; *B. caeruleus* venom, NNV; *N. naja* venom, ECV; *E. carinatus* venom and DRV; *D. russelii* venom.

**Figure 4:**
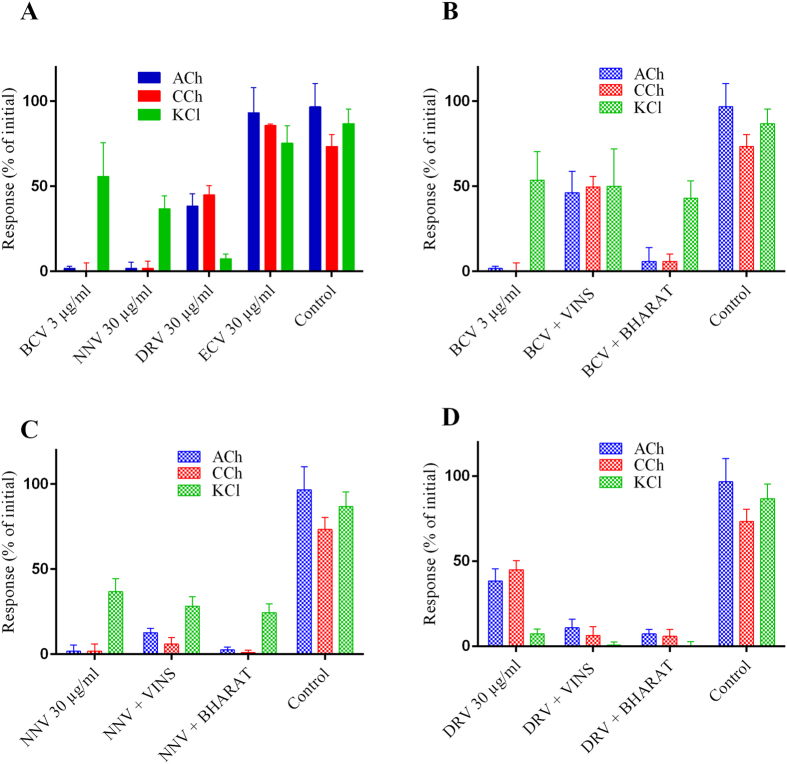
Response to acetylcholine (ACh), carbachol (CCh) and potassium chloride (KCl) for venom alone (**A**) and with five times recommended doses of VINS and BHARAT antivenoms; (**B**) *B. caeruleus* (3 μg/ml) venom, (**C**) *N. naja* (30 μg/ml) venom and (**D**) *D. russelii* (30 μg/ml) venom on indirect twitches of the chick biventer cervicis nerve–muscle preparation. BCV; *B. caeruleus* venom, NNV; *N. naja* venom, ECV; *E. carinatus* venom and DRV; *D. russelii* venom.

*B. caeruleus* venom (3 μg/ml; N = 3) caused rapid inhibition (t_90_ = 33.5 min) of nerve-mediated twitches in the chick biventer preparation, while *N. naja* (N = 4) and *D. russelii* (N = 3) venoms only caused partial inhibition of nerve-mediated twitches at a concentration of 3 μg/ml (Fig. 3A). *E. carinatus* venoms (N = 3) did not cause inhibition of nerve-mediated twitches at any dose. For the increased concentration of 30 μg/ml, *N. naja* (t_90_ = 29.5 min) and *D. russelii* (t_90_ = 44.0 min) venom did inhibit nerve-mediated twitches (Fig. 3A, Table 4), confirming that these venoms were less neurotoxic than krait venom. *B. caeruleus* and *N. naja* venom significantly inhibited responses to exogenous ACh and CCh (Fig. 4A), while having no significant effect on the response to KCl, indicating the presence of postsynaptic neurotoxins in *B. caeruleus* venom, but not excluding pre-synaptic activity. *D. russelii* venom only partially inhibited the response to exogenous ACh and CCh (Fig. 4).

### Neutralization of neurotoxicity of *B. caeruleus, N. naja* and *D. russelii* venoms

Addition of the recommended concentration of VINS and BHARAT antivenom did not prevent the inhibition of twitches induced by any of the venoms, so five times the recommended concentration of the antivenoms was used as per previous studies^20^. At this concentration VINS antivenom effectively prevented *B. caeruleus* venom induced twitch inhibition compared to no effect with BHARAT antivenom (Fig. 3B, Table 4). There was partial recovery of the response to exogenous ACh and CCh with VINS and BHARAT antivenoms following *B. caeruleus* venom (Fig. 4B). However, both VINS and BHARAT antivenoms had minimal effect in preventing *N. naja* venom induced twitch inhibition (Fig. 3C, Table 4), and no effect in preventing the partial inhibition of twitches induced by *D. russelii* venom (Fig. 3D, Table 4).

### Lethality neutralization studies

Lethality and neutralization studies for the antivenoms were investigated by calculating the median lethal dose (LD_50_) and the median effective dose (ED_50_) values in mice. LD_50_ values of *D. russelli* and *B. caeruleus* venoms were less than that of *E. carinatus* and *N. naja* venom in mouse experiments (Table 5). VINS antivenom appeared to be slightly more effective in the neutralization of lethality induced by *D. russelli* and *B. caeruleus* venoms compared to BHARAT antivenom, but this was not statistically significant (Table 5). VINS was almost twice as effective against *N. naja* venom compared to BHARAT, which was almost statistically significant. There was no numerical difference in the ED_50_ values for *E. carinatus* venom (Table 5). The ER_50_ for *D. russelli* venoms was 2.06 for VINS compared to 1.24 for BHARAT; for *E. carinatus* venom was 2.79 for VINS compared to 2.82 for BHARAT; for *B. caeruleus* was 3.92 for VINS compared to 2.93 for BHARAT; and for *N. naja* was 4.32 for VINS compared to 2.42 for BHARAT.

## Discussion

This study has shown that VINS antivenom has a higher protein content and overall a greater *in vitro* efficacy against the medically important effects of most snake venoms in Sri Lanka, compared to BHARAT antivenom. VINS antivenom performed better against the clinically relevant effects of three snakes, being more efficacious against the *in vitro* procoagulant activity of *D. russelii* and *E. carinatus* venoms, and the neurotoxic effects of *B. caeruleus* venom. A concerning finding was that the lethality and ED_50_ studies did not reflect these findings. The ED_50_ for the two antivenoms was not statistically significantly different for *D. russelii*, *B. caeruleus* and *E. carinatus*, in contrast to VINS being more efficacious against clinically relevant effects–coagulopathy and neurotoxicity. The study also found some variation in the protein content and efficacy of the different VINS antivenom batches from 2008 to 2012, with decreased protein content and reduced capacity of antivenoms binding venom antigens, for antivenoms manufactured in 2008 and 2010.

In a previous study of Russell’s viper envenoming where antivenom concentrations were measured in 86 patients after administration of 10 vials of antivenom, the median antivenom concentration was 2.2 mg/ml a median of two hours after administration of 10 vials of antivenom^21^. Tables 2, 3, 4 show that this concentration is sufficient for complete binding of all four venom antigens, neutralisation of procoagulant effects and neutralisation of neurotoxicity by VINS antivenom. However, this was not true for BHARAT antivenom in which larger concentrations were required to bind the venom antigens and neutralise the procoagulant effects of *E. carinatus* venom. Both antivenoms required higher concentrations to neutralise the procoagulant effect of *E. carinatus* venom suggesting they are not as efficacious against this venom, although this concentration appears to be sufficient for VINS antivenom. The inferior efficacy of BHARAT antivenoms was consistent with the lower protein content in these antivenoms.

An unusual finding was that BHARAT antivenom had a significantly higher venom antigen binding capacity for *N. naja* venom than VINS antivenom. However, BHARAT antivenom was less effective in neutralising the neurotoxic effects of *N. naja* venom and had a higher ED_50_ compared to VINS. This differed to the other venoms and suggests that BHARAT antivenom has a higher titre to cobra venom antigenic components that may not be toxic. One study found that BHARAT antivenom was ineffective against the neurotoxicity of Pakistani cobra (*N. naja*) and Pakistani krait (*B. sindarus*) at similar concentrations^22^. Unfortunately this study did not test other antivenoms and concluded that antivenom in general was ineffective against Pakistani snake neurotoxicity^22^. A more recent study found that higher titres of three different antivenoms (Indian, Thailand and Taiwan) were required to neutralise the neurotoxic effects^23^. Another study found that VINS antivenom bound more avidly to Indian compared to Sri Lankan cobra venom, also suggesting possible geographical variability in the venoms, particularly the neurotoxic activity^24^.

A limitation of the study was that only the most recent batches of BHARAT antivenoms were available to be tested and only multiple vials from one batch of each type of antivenom was available. However, the two recent batches of BHARAT were inferior to the recent batches of VINS tested against the important clinical effects of the medically important snakes in Sri Lanka and a direct comparison between batches with multiple vials found VINS to be statistically significantly more efficacious than BHARAT antivenoms. In addition, the variability within these two batches was much greater for BHARAT than for VINS. We have previously shown that expired antivenoms up to 10 years old, formulated as liquids, have lost minimal activity, even after extended periods at room temperature^25^. Antibodies in the solid form would be expected to be as least as stable.

The units of antivenom differed for the lethality studies compared to the *in vitro* studies because the lethality studies are dosed on mouse body weight. This is another reason that lethality studies are problematic because it is difficult to relate μg/g body weight to the amount of venom in human bites. For the binding and *in vitro* studies we used concentrations measured in human snake envenoming cases. The difference in units did not affect the conclusions of the study because the relative efficacy of the two antivenoms differed in lethality studies compared to the *in vitro* studies. In addition, the ER_50_ was also calculated to provide another comparison without units.

Another limitation was that the study did not test the efficacy of the antivenom against other known venom effects, such as myotoxicity or nephrotoxicity^26^. There are no well tested methods of assessing nephrotoxicity^26^. One study reports only minor changes in renal function in an *in vivo* murine model, evidenced by protein and erythrocytes in urine, but not renal histology or measurements of creatinine. Myotoxicity is only a minor clinical problem in envenoming by all of these snakes^27^. One study reported an unusually high early increase in creatine kinase in mice 3 hours post-injection of *D. russelii* venom, which is too early to be due to systemic myotoxicity and not consistent with a previous *in vivo* examination of systemic myotoxicity due to snake venoms in rats^28^. Coagulopathy is the most important clinical effect in Sri Lankan Russell’s viper and saw-scaled viper envenoming, so testing the efficacy of antivenom against the procoagulant effect is most appropriate. Other haemotoxic venom effects were also not tested, such as haemorrhagic effects and platelet toxicity. Although haemorrhagic effects are important for *E. carinatus* neither of the other haemotoxic effects are important in *D. russelii* bites.

Neurotoxicity is the most important clinical effect in *B. caeruleus* envenoming^27^, so testing antivenom against neurotoxicity was most appropriate. In addition, we tested the efficacy of the antivenoms to bind to venom antigenic components as a general assessment of antivenom efficacy. There was good correlation between binding efficacy and efficacy against medically important clinical effects, except for cobra neurotoxicity where BHARAT antivenom was found to bind more effectively. VINS antivenom had excellent binding efficacy for *D. russelii*, and more recent vials had better binding efficacy for *B. caeruleus* and *E. carinatus*. The binding efficacy was statistically significantly better for VINS for all snakes except *E. carinatus*. VINS antivenoms were also more efficacious in neutralising procoagulant effects for *D. russelii* and *E. carinatus*, and neurotoxic effects for *B. caeruleus*. Neither antivenom was able to neutralise the neurotoxic effects of *D. russelii* venom. Although neurotoxicity occurs in about half of *D. russelii* bites in Sri Lanka it is rarely life-threatening^29^. Neurotoxicity is only reported for Russell’s viper bites in Sri Lanka and Southern India^30^, so the venoms used in making the Indian antivenoms may not contain these neurotoxins^31^. This supports assessing antivenoms using tests of *in vitro* efficacy against clinically relevant toxicity.

In contrast to efficacy as assessed by *in vitro* binding and neutralisation efficacy, efficacy assessed by traditional ED_50_ testing against lethality in mice, was not statistically significant between the antivenoms for any of the venoms. The relative efficacy of the two antivenoms based on ED_50_ values was not consistent with any of the testing against important clinical effects in any of the snakes, suggesting that relying on such testing is problematic. The reason for this is that death in animals (e.g, mice in this study) could be due to a range of toxicities including some clinically irrelevant toxic effects important in human envenoming. It is entirely possible that post-synaptic neurotoxins or early cardiovascular collapse are major causes of lethality in rodents^32^, but are far less important in humans, in which presynaptic neurotoxins and procoagulant toxins are more prominent. A recent study has found that the procoagulant toxins in snake venoms have different effects on human and animal plasmas, making interpretation of efficacy of antivenom in rodent models problematic^12^. Based on the results of the current studies it would appear to be more appropriate to use clinically relevant *in vitro* studies of antivenoms against venom effects, and great care should be taken when interpreting *in vivo* animal models. However, further work is required on other snakes worldwide to confirm our findings for all antivenoms.

VINS antivenom appears to be the more efficacious compared to BHARAT antivenom. A dose of 10 vials is sufficient to bind all free venom antigens from these four snakes for venom concentrations found in patients with human envenoming. In addition, this dose was also able to neutralise the procoagulant effects *in vitro* of *D. russelii* venom and *E. carinatus* venom, and the neurotoxic effects of *B. caeruleus*. In contrast, the lethality studies did not appear to provide as useful an assessment or comparison of the efficacy of the two antivenoms.

## Methods

### Materials

Indian polyvalent snake antivenom was obtained from VINS Bioproducts Limited (Hyderabad, Andra Pradesh, India) and from BHARAT Serum and Vaccines Limited (Mumbai Maharashtra, India). Details of the antivenoms tested are given in Supplementary Table 1. All antivenoms were reconstituted according to the manufacturer’s instructions. Russell’s viper (*D. russelii*), common cobra venom (*N. naja*), Saw-scaled viper (*E. carinatus*) and common krait (*B. caeruleus*) venoms were collected in Sri Lanka. Stock solutions of venom was prepared as 1 mg/mL in 50% Glycerol and stored at −20 °C.

Bradford reagent (Bio-Rad, Catalogue # 500–0205) and Bovine Gamma Globulin (Bio-Rad, Catalogue # 500–0208) were used for protein quantification. Tris-buffered saline (TBS) was used to make up dilutions of antivenom for neutralization of *D. russelii* and *E. carinatus* venom procoagulant activity studies. Fresh frozen plasma was obtained from the Australian Red Cross and aliquots of 10 mL were thawed at 37 °C. Tetramethylbenzidine (TMB) from Sigma, bovine serum albumin (BSA) from Bovogen, Australia and Streptavidin-conjugated horseradish peroxidase (Streptavidin HRP) from Millipore Chemicon were used for the binding studies. Blocking solution is 0.5% BSA in phosphate buffered saline (PBS). Washing solution is 0.02% TWEEN 20 in PBS. Polyclonal monovalent rabbit IgG to Russell’s viper venom was obtained by injection of rabbit with *D. russelii* venom followed by purification of the serum on a Protein G-Sepharose column and was carried out at the Western Australian Institute of Medical Research. Polyclonal monovalent rabbit IgG to *E. carinatus*, *N. naja* and *B. caeruleus* venom were obtained by injection of rabbits with the corresponding venoms, followed by purification of the serum on a Protein G-Sepharose column and was carried out at the Faculty of Medicine and Allied sciences, Rajarata University, Sri Lanka. Rabbit IgG antibodies were biotinylated using EZ-Link Sulfo-NHS-LC-Biotin (Pierce # 21335).

Binding and neutralisation studies of *D. russelii* venom were undertaken at venom concentrations measured in cases of human envenoming and taken as 1.7 μg/ml, the 97^th^ percentile of venom concentrations in a previous study of 257 patients with pre-antivenom venom concentrations ranging from 0.0033 to 14.8 μg/mL^21^. For *E. carinatus* venom the venom concentration used was 0.5 μg/mL based on the maximum concentrations measured in one study of *Echis ocellatus* envenoming^33^. For *N. naja* and *B. caeruleus* venoms the venom concentrations was again taken as the 97^th^ percentile which was 1.4 μg/ml for *N. naja* and 0.15 μg/ml for *B. caeruleus* based on nine envenomed patients from Sri Lanka for each venom. Only one batch of antivenom from each manufacturer were compared statistically -VINS (01011/10–11; 2010) and BHARAT (A5311006; 2011Jan)–because multiple vials were available.

### Protein quantification

Quantification of the protein content in each antivenom was undertaken using the Bradford protein assay method^34^ Inter- and intra-batch protein quantification was carried out for all 36 vials of antivenom. Bradford reagent (150 μl) was added to a solution of antivenom in PBS (150 μl). After 10 minutes absorbance at 595 nm was measured on a Bio-Tek ELx808 plate reader. Concentrations of proteins were calculated with reference to a standard curve based on bovine gamma-globulin. Samples were measured at three dilutions.

### Venom-antivenom binding studies

The following antivenom vials were used for antivenom venom binding studies–VINS 1061; VINS 01011/10–11; VINS 01013/10–11; VINS 01023/10–11; VINS 01024/10–11; VINS 01AS11112; VINS 01AS11114; BHARAT A5311006; BHARAT A5311013; BHARAT A5311014. Solutions of increasing concentrations of antivenom (0 to 4.3 mg/mL for *E. carinatus* venom and 0 to 17.1 mg/mL for *D. russelii, N. naja* and *B. caeruleus* venom) in blocking solution (0.5% Bovine Serum Albumin in PBS) were incubated with venom (*D. russelii* 1.7 μg/mL, *E. carinatus* 0.5 μg/mL*, N. naja* 1.4 μg/mL and *B. caeruleus* 0.15 μg/mL) for one hour at room temperature. Unbound venom antigens were detected using a sandwich enzyme immunoassay (EIA) as previously described^35^. In brief, Greiner Microlon 96-well high-binding plates were coated with the four different monovalent rabbit anti-snake venom IgGs (1 μg/mL) in carbonate buffer (50 mM, pH 9.6), kept at room temperature for 1 h and then at 4 °C overnight. The plates were then washed once with PBS containing 0.02% TWEEN 20 and 300 μL of blocking solution of 0.5% BSA in PBS was added. After 1 h the plates were washed again, and 100 μL of venom–antivenom mixture was applied, after first diluting appropriately in blocking solution applied as (1:400 for *D. russelii,* 1:25 for *E. carinatus,* 1:140 for *N. naja,* 1:15 for *B. caeruleus*) dilutions in blocking solution. The plates were allowed to stand for 1 h and then washed three times. Next, biotinylated anti-snake venom IgG (*D. russelii* 0.5 μg/mL, *E. carinatus* 8 μg/mL*, N. naja* 0.12 μg/mL and *B. caeruleus* 1 μg/mL in blocking solution) was added. After standing for a further hour the plates were washed again. Streptavidin-horseradish peroxidase (100 μL, 0.1 μg/mL in blocking solution) was added and left for 1 h. The plate was then washed three times and 100 mL of TMB reagent added and colour allowed to develop for 3.5 min. The reaction was stopped by the addition of 50 mL of 1 M H_2_SO_4_. All samples were measured in triplicate, and the averaged absorbance converted to a concentration of the venom of interest by comparison with a standard curve based on serial dilutions of venom.

### Neutralization of procoagulant activity of D. russelii and E. carinatus venom

The same antivenom vials used for *D. russelii* and *E. carinatus* venom binding studies were used for neutralization studies. The ability of antivenom to neutralise the procoagulant activity of *D. russelii* and *E. carinatus* venoms was measured using the turbidimetric method^13^. Solutions of increasing concentrations of antivenom in TBS (0 to 1 mg/mL for *D. russelii* venom and 0 to 30 mg/mL for *E. carinatus* venom) were incubated with 1.7 μg/mL of *D. russelii* venom or 0.5 μg/mL of *E. carinatus* venom for 30 min at 37 °C in a 96 well plate. Fresh frozen plasma (100 μl) containing 40 μl of 0.4 M CaCl_2_/mL and venom-antivenom solution were added simultaneously to each well using a multichannel pipette. After a 5-second shaking step, the optical density at 340 nm was monitored every 30 s for 20 min. The clotting time was defined as the time until the rapid increase in absorbance, as calculated by Gen5 software (supplied with the Biotek ELx808 plate reader).

### *In vitro* neurotoxicity studies

VINS (01AS11114) and BHARAT (A5311014) antivenom batches were used to investigate the neutralisation of the neurotoxic effects of Russell’s viper (*D. russelii*), Common cobra (*N. naja*) and Indian krait (*B. caeruleus*) venoms. Chicks (4 to 10-day-old males) were killed by CO_2_ inhalation and exsanguination, and the two biventer cervicis muscles were removed from the back of the neck. Each muscle was attached to a wire tissue holder and placed in a 5 mL organ bath filled with physiological salt solution with the following composition (mM): NaCl, 118.4; NaHCO_3_, 25; glucose, 11.1; KCl, 4.7; MgSO_4_, 1.2; KH_2_PO_4_, 1.2 and CaCl_2_, 2.5. The organ baths were bubbled with carbogen (95% O_2_, 5% CO_2_) and maintained at a temperature of 33–34 °C under a resting tension of 1 g. Motor nerves were indirectly stimulated every 10 s (0.2 ms duration) at supramaximal voltage using a Grass S88 stimulator. The tissues were equilibrated for 10–15 min after which d-tubocurarine (10 μM) was added, and the subsequent abolition of twitches confirmed the selective stimulation of the motor nerves. The tissues were then washed repeatedly until twitch height was restored. Contractile responses to acetylcholine (ACh; 1 mM for 30 s), carbachol (CCh; 20 μM for 60 s), and KCl (40 mM for 30 s) were measured in the absence of stimulation^36^. At the conclusion of the experiment, responses to ACh, CCh, and KCl were measured again. Each of the four venoms at two concentrations (3 and 30 μg/mL) were initially added to the organ bath without antivenom to determine the neurotoxic potency of each venom. Only *B. caerulus* venom had significant neurotoxicity at concentrations seen in human envenoming (3 μg/mL). Neurotoxicity potency was measured as the t_90_ which is the time required to cause 90% inhibition of the initial twitch height, for a given concentration of venom, expressed as a mean + /− SD. For some venoms, where the twitch height inhibition for a particular venom/treatment did not reach 90%, the time to maximum twitch inhibition observed in all tissues of that treatment/venom group was considered for all the groups for comparison.

Antivenom was added to the bath and incubated for 10 min. *D. russelii* (30 μg/mL), *N. naja* venom (30 μg/mL) or *B. caeruleus* venom (3 μg/mL) was then added and left in contact with the tissue for 90 min. The antivenom concentration used was based on the manufacturer’s instructions (1 ml of antivenom neutralises 0.6 mg *N. naja* venom, 0.45 mg *B. caerulus* venom and 0.6 mg of *D. russelii* venom). Experiments were done for both the recommended dose and five times the recommended dose.

### Lethality neutralization studies

Lethality neutralization studies on antivenoms were tested by calculating the median lethal dose (LD_50_) and the median effective dose (ED_50_) values in mouse experiments. ICR (Institute of Cancer Research) mice, both sexes, weighing 18–20 g were used for all experiments. The following antivenom vials VINS0101110/11 and Bharat A5311006 were used for lethality studies. For this study, 250 mg/ml solutions of the antivenoms were prepared for venom-neutralization studies which was higher than the manufacturer’s recommended concentration because the recommended concentration was too dilute and therefore too large a volume for administration to mice

Assessment of the LD_50_ and ED_50_ experiments followed the methods described by the World Health Organisation (WHO) (2010)^9^. After dose ranging studies, varying doses of venom or venom and antivenom mixtures were injected to multiple groups of five mice in both LD_50_ and ED_50_ experiments. In all ED_50_ studies, five times the LD_50_ of the respective venom was mixed with varying amounts of antivenom and incubated at 37 °C for 30 min before injection. All injections were intravenous to the tail veins and were made to a final constant volume of 250 μl by adding normal saline. In both LD_50_ and ED_50_ studies, death/survival rates were recorded for 48 hours. The ED_50_ values were expressed in μg of antivenom per g body weight of mouse (μg/g) to neutralize the challenge dose of venom. The median effective ratio (ER_50_) was also calculated using the following formula,


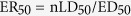


where n is the number of LD_50_ doses given (5 in this study).

### Ethics Approvals

Ethical approval for the chick experiments was obtained from the Monash University Animal Ethics Committee 2012/008. All animal experiments were conducted in the Animal House, Faculty of Medicine and Allied Sciences, Rajarata University of Sri Lanka according to the Council for International Organizations of Medical Sciences (CIOMS) guidelines on animal experimentation^37^. Animal ethics clearance for the study was obtained from the Ethics review committee, Faculty of Medicine and Allied Sciences, Rajarata university of Sri Lanka ERC 2012/038.

### Analysis of results

Standard curves for enzyme immunoassays and calculations of EC_50_ were fitted by non-linear regression. For the *in vitro* neurotoxicity data the twitch heights were analysed by one way ANOVA followed by Bonferroni post-hoc tests, and the time to a reduction in twitch heights by Kruskal-Wallis test followed by Dunn’s multiple comparison test. Standard error of the mean (SEM), standard deviations (SD) and 95% confidence intervals (95% CI) were all calculated for parametric and non-parametric data respectively. The difference in protein content between vials from one batch of each of the two antivenoms was compared using an unpaired t-test. Log EC_50_ values were compared using the extra sum of the squares F test in Prism when comparing one antivenom from each manufacturer. The 95% confidence intervals of the ED_50_ values were compared to determine if the ED_50_ values were significantly different. Statistical significance was set at p < 0.05. All analyses and graphics were done in GraphPad Prism version 6.03 for Windows, GraphPad Software, San Diego California USA, www.graphpad.com, except for the calculation of the LD_50_ and ED_50_ which was done using the probit method^38^ using SPSS statistical software version 20.0.

## Additional Information

**How to cite this article**: Maduwage, K. *et al.* Efficacy of Indian polyvalent snake antivenoms against Sri Lankan snake venoms: lethality studies or clinically focussed *in vitro* studies. *Sci. Rep.* **6**, 26778; doi: 10.1038/srep26778 (2016).

## Supplementary Material

Supplementary Information

## Figures and Tables

**Table 1:** The dry powder weight of antivenom, the percentage of proteins per mg of antivenom and the amount of protein per vial in VINS and BHARAT antivenoms.

Vial Type	Dry powder wt. (mg/vial)	Percent protein	Protein content/vial (mg)
VINS2000 (1054)	800	30.7%	246
VINS2008 (1061)	614	26.4%	162
VINS2010* (01011/10–11)	658 (535–815)	30.3% (23–36)	198 (157–238)
VINS2011 (01AS11112)	801	40.9%	328
VINS2012 (01AS11114)	798	62.7%	500
BHARAT2011* (A5311006)	390 (155–510)	25.2% (23–27)	98 (39–125)

^*^The median value and range is reported for VINS2010 and BHARAT2011 based on testing 10 vials from each batch.

**Table 2:** Median effective concentration (EC_50_) with 95% confidence intervals for VINS and BHARAT antivenoms binding of 1.7 μg/mL of *D. russelii*, 0.5 μg/mL of *E. carinatus*, 1.4 μg/mL of *N. naja* and 0.15 μg/mL of *B. caeruleus* venom antigens.

Vial Type	Median effective concentration (EC_50_) μg/mL			
	1.7 μg/mL of *D. russelii* venom	0.5 μg/mL of *E. carinatus* venom	1.4 μg/mL of *N. naja* venom	0.15 μg/mL of *B. caeruleus*venom
VINS2008 (1061)	324 (133–791)	165 (160–170)	253 (231–277)	131 (108–157)
VINS2010 (01011, 01013, 01023, 01024)	553 (237–1289)	165 (156–172)	336 (325–347)	97 (85–110)
VINS2011 (01AS11112)	248 (103–599)	158 (157–159)	281 (238–332)	155 (130–186)
VINS2012 (01AS11114)	262 (100–686)	166 (163–169)	142 (133–152)	42 (31–56)
BHARAT 2011Jan (A5311006)	1371 (956–1965)	187 (168–208)	70 (53–91)	157 (121–205)
BHARAT 2011Apr (A5311013, A5311014)	1051 (837–1319)	167 (165–170)	59 (53–66)	132 (111–158)

**Table 3:** Median effective concentrations (EC_50_) of antivenom in neutralising the procoagulant activities of 1.7 μg/mL of *D. russelii* venom and 0.5 μg/mL of *E. carinatus* venom in human plasma for VINS versus BHARAT antivenoms.

Vial Type	Median effective concentration (EC_50_) μg/mL	
	1.7 μg/mL of *D. russelii* venom	0.5 μg/mL of *E. carinatus* venom
VINS2008 (1061)	48 (45–52)	645 (443–939)
VINS2010 (01011, 01013, 01023, 01024)	60 (44–81)	357 (87–1458)
VINS2011 (01AS11112)	42 (37–48)	367 (169–793)
VINS2012 (01AS11114)	49 (40–60)	330 (142–766)
BHARAT 2011Jan (A5311006)	176 (149–207)	6906 (4858–9817)
BHARAT 2011Apr (A5311013, A5311014)	84 (67–105)	859 (410–1800)

**Table 4:** Inhibition of indirect stimulation of chick biventer cervicis nerve-muscle preparations (time to 90% inhibition [t_90_]; mean and standard deviation [SD]) by *B. caeruleus, N. naja* and *D. russelii* venoms and the effect of preventing twitch height inhibition by VINS and BHARAT antivenoms.

t_90_ (mean [SD])	Venom alone	Venom + VINS antivenom	Venom + BHARAT antivenom
*B. caeruleus* (3 μg/ml)	33.5 [2.5]	Delayed (see Fig. 3B)	40.0 [2.6]
*N. naja* (30 μg/ml)	29.5 [5.1]	Delayed (see Fig. 3C)	52.0 [9.3]
*D. russelii* (30 μg/ml)	44.0 [3.5]	61.5 [11.9]	57.5 [9.5]

Five times the recommended amount of antivenom was used for each antivenom.

**Table 5:** Lethality dose 50 (LD_50_) and effective dose 50 (ED_50_) of VINS and BHARAT antivenoms for *D. russelii*, *E. carinatus*, *N. naja* and *B. caeruleus* venom.

Venom	LD_50_μg/g body weight of mice (95% confidence intervals)	ED_50_μg/g body weight of mice (95% confidence intervals) for VINS 2010	ED_50_μg/g body weight of mice (95% confidence intervals) for BHARAT 2011 (Jan)
*D. russelii*	0.102 (0.075–0.121)	0.248 (0.175–0.375)	0.412 (0.310–0.505)
*E. carinatus*	0.664 (0.519–0.806)	1.188 (0.862–1.666)	1.177 (0.690–2.112)
*N. naja*	0.665 (0.482–0.978)	0.770 (0.484–1.052)	1.375 (0.986–1.632)
*B. caeruleus*	0.196 (0.148–0.251)	0.250 (0.090–0.520)	0.334 (0.212–0.590)

^*^ED 50 is calculated for neutralizing doses of five times LD_50_ values of each venom.
