# Supplementary material for: Efficacy of Indian polyvalent snake antivenoms against Sri Lankan snake venoms: lethality studies or clinically focussed in vitro studies
Source: Sci Rep. 2016 May 27;6:26778. doi: 10.1038/srep26778 (PMC4882578; doi:10.1038/srep26778)
Supplement: Supplementary Information [file srep26778-s1.pdf]

Efficacy of Indian polyvalent snake antivenoms against Sri Lankan snake venoms: lethality studies or clinically focussed *in vitro* studies.

Kalana Maduwage[1,2], Anjana Silva[3,4], Margaret A O'Leary[1], Wayne C Hodgson[3], Geoffrey K Isbister[1,2]

Supp Figure 1. Plots of percent free (unbound) venom versus the logarithm of the antivenom concentration for the one batch of antivenom from VINS and one from BHARAT showing the binding capacity for (A) *D. russelii*, (B) *E. carinatus*, (C) *N. naja* and (D) *B. caeruleus* venoms.

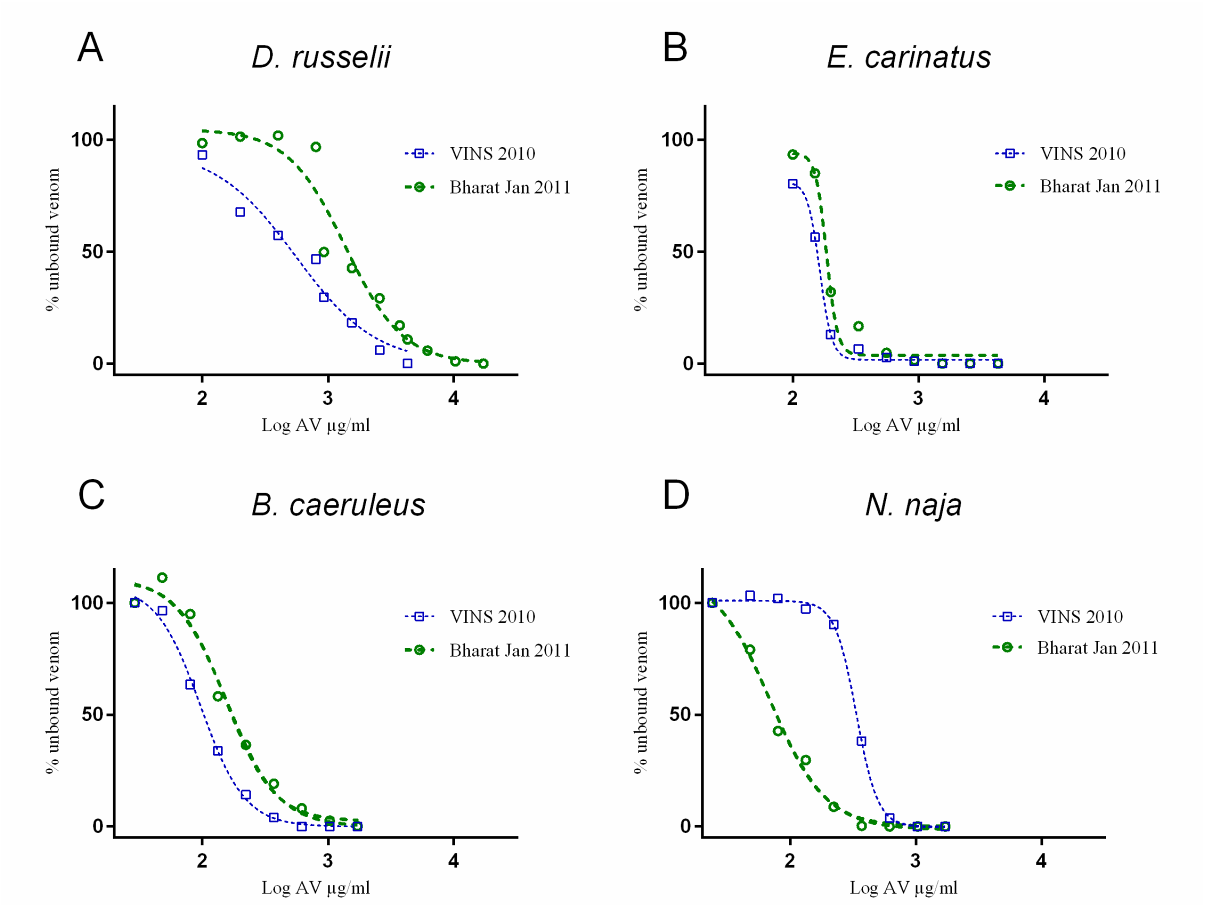

Supp Figure 2. The effect of one batch of VINS and one batch of BHARAT antivenom on delaying the procoagulant activities of 1.7µg/mL of *D. russelii* (A), 0.5µg/mL of *E. carinatus* (B) venom on human plasma.

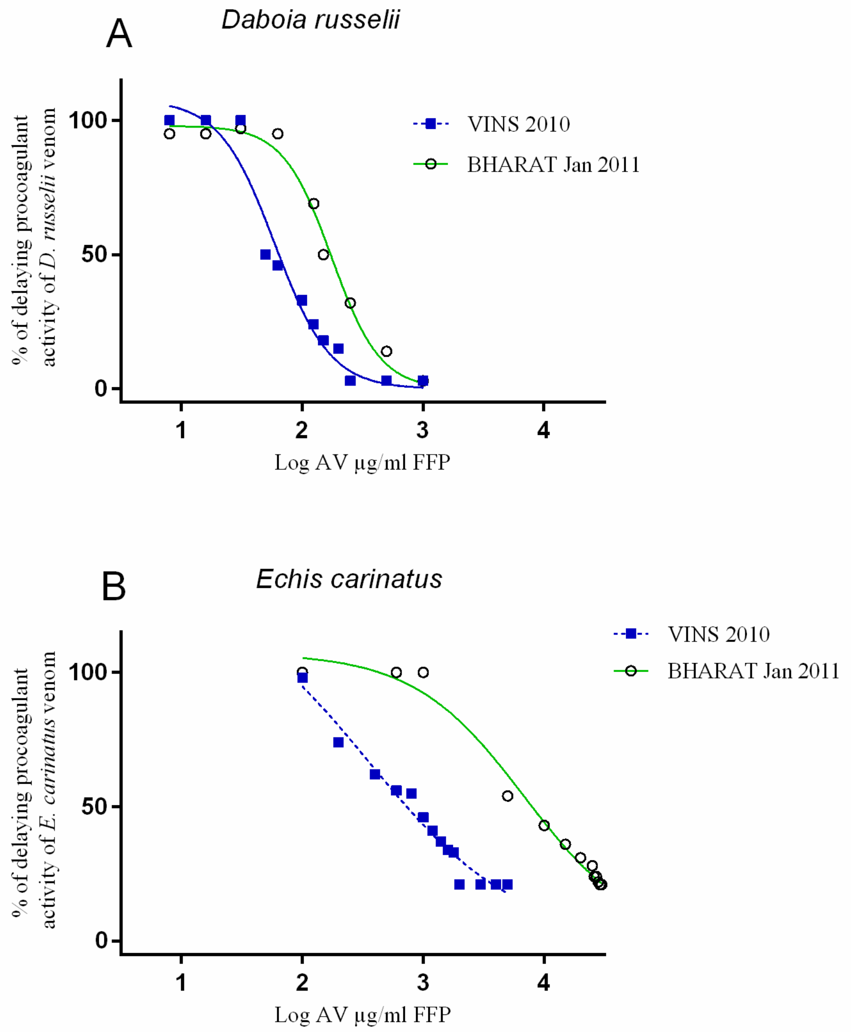

Supplementary Table 1. Details of VINS and BHARAT antivenoms used for the experiments.

Company	Batch number	Manufacture date	Expiry date	Number of vials
VINS	1054	Sep 2000	Aug 2004	1
VINS	1061	Oct 2008	Sep 2012	1
VINS	01011 / 10-11	May 2010	Apr 2014	10
VINS	01013 / 10-11	May 2010	Apr 2014	1
VINS	01014 / 10-11	May 2010	Apr 2014	1
VINS	01015 / 10-11	May 2010	Apr 2014	1
VINS	01021 / 10-11	May 2010	Apr 2014	1
VINS	01022 / 10-11	May 2010	Apr 2014	1
VINS	01023 / 10-11	May 2010	Apr 2014	1
VINS	01024 / 10-11	May 2010	Apr 2014	1
VINS	01042 / 10-11	Jul 2010	Jun 2014	1
VINS	01057 / 10-11	Sep 2010	Aug 2014	1
VINS	01058 / 10-11	Sep 2010	Aug 2014	1
VINS	01AS11112	Dec 2011	Nov 2016	1
VINS	01AS11114	Jan 2012	Dec 2016	1
BHARAT	A5311006	Jan 2011	Dec 2014	10
BHARAT	A5311013	Apr 2011	Mar 2015	1
BHARAT	A5311014	Apr 2011	Mar 2015	1
